# Sleep Health, Depression, Pain and Quality of Life among Older Adults during the First Months of the Covid-19 Pandemic

**DOI:** 10.1093/geroni/igab046.3134

**Published:** 2021-12-17

**Authors:** Rebecca Lorenz, Devita Stallings, Janice Palmer, Helen Lach

**Affiliations:** 1 University at Buffalo, Buffalo, New York, United States; 2 Saint Louis University, St. Louis, Missouri, United States; 3 Webster University, St. Louis, Missouri, United States; 4 Saint Louis University, Saint Louis, Missouri, United States

## Abstract

To slow the spread of Covid-19, many states instituted restrictions on group size for religious services, exercise, and social engagement. We are beginning to understand the effect of these mandates on older adults. The purpose of this study was to examine the relationships between sleep health, depression, pain, and quality of life (QOL) among older adults during the initial months of the Covid-19 pandemic. Older adults completed an anonymous online survey to collect data including personal characteristics, behaviors, and health conditions during May-September 2020. Sleep Health was assessed with a survey of satisfaction, timing, efficiency, and duration of sleep along with daytime alertness. Pearson correlations were used to explore relationships between age, education, socioeconomic status, pain, depression, and QOL. Participants (N=509) were primarily female (n=392, 77%), white (n=466; 92%), college educated (n=471, 93%) and with a mean age of 75.6 years (SD=5.0; range 63-93 years). Mean Sleep Health score was 7.4 (SD=2.1; range 0-10). Higher (better) Sleep Health scores were associated with education (r=.15, p<01) and socioeconomic status (r=.17, p<.01) and lower scores with depression (r= -.35, p<.01), pain (r= -.23, p<.01), and QOL (r= -.26, p<.01). Poorer Sleep Health among older adults during the initial months of the pandemic were associated with depression, pain, and reduced QOL. Sleep, depression, and pain have reciprocal relationships that may have lasting consequences on physical and mental health among older adults. These findings suggest that poor sleep health should be identified and treated to improve QOL among older adults.

